# Shedding of SARS-CoV-2 for 85 Days in COVID-19 Patients With Type 2 Diabetes Mellitus and Lung Metastasis: A Case Report

**DOI:** 10.3389/fmed.2022.828819

**Published:** 2022-03-28

**Authors:** Xiaoheng Wu, Min Shen, Hui Quan, Xianqin Zhang, Fengcheng Xu, Juan Li, Miao He, Dongmei Pan, Ling Cao, Changwu Yue, Tianhu Liu, Xu Jia

**Affiliations:** ^1^Yan'an Key Laboratory of Microbial Drug Innovation and Transformation, School of Basic Medicine, Yan'an University, Yan'an, China; ^2^Non-coding RNA and Drug Discovery Key Laboratory of Sichuan Province, Chengdu Medical College, Chengdu, China; ^3^The 2nd Affiliated Hospital of Chengdu Medical College, Nuclear Industry 416 Hospital, Chengdu, China; ^4^School of Basic Medical Sciences, Chengdu Medical College, Chengdu, China; ^5^The 3rd Affiliated Hospital of Chengdu Medical College, Pidu District People's Hospital, Chengdu, China; ^6^Institute of Blood Transfusion, Chinese Academy of Medical Sciences, Chengdu, China; ^7^Public Health and Clinical Center of Chengdu, Chengdu, China

**Keywords:** SARS-CoV-2, asymptomatic, reinfection, type 2 diabetes, lung-occupying site

## Abstract

**Background:**

COVID-19 (coronavirus disease 2019) caused by severe acute respiratory syndrome coronavirus 2 (SARS-CoV-2) seriously endangers people's lives. The variation in SARS-CoV-2 makes the research and development of vaccines and specific drugs particularly important. However, the prevention and diagnosis of COVID-19 cannot be underestimated in the control of the epidemic.

**Case Presentation:**

We introduced a 65-year-old female patient who was diagnosed with COVID-19. The SARS-CoV-2 nucleic acid test result of this patient was positive again during treatment. It took 85 days from the first symptom to the final cure. According to the known reports, she is currently the patient with the longest virus shedding in Sichuan Province, China. Due to the patient's special condition, she was treated in four hospitals before and after, and she was diagnosed with type 2 diabetes mellitus (T2DM) and right lung metastatic adenocarcinoma. We fully introduced the patient's epidemiological history, diagnosis, testing, and treatment process. The patient was finally discharged from the hospital under the treatment of antiviral, hypoglycaemic, anti-anxiety, and a combination of Chinese and Western medicine.

**Conclusions:**

The epidemic is still rampant, and we should not relax our efforts in the prevention and control of viruses. For the elderly, especially those who are suffering from complications or vulnerable to diseases, it is recommended to extend the observation time. Additionally, medical workers should pay attention to the mental state of patients.

## Introduction

COVID-19 is an acute respiratory infectious disease caused by SARS-CoV-2 infection that can be transmitted by humans or other intermediate hosts (1). The severity of SARS-CoV-2 infection is determined by both viral infection and host response, which is similar to SARS-CoV. The patients often have symptoms such as fever, dry cough, dyspnea, nausea, and could develop acute respiratory distress syndrome (ARDS) (2). Patients with COVID-19 often have comorbidities. Common complications occur in the kidney, respiratory system and whole body, and complications in gastrointestinal tract and nervous system have also been reported (3). Diabetes mellitus (primary T2DM) is one of the major complications of COVID-19 patients. It would increase the incidence of other complications, including ARDS and multiple organ failure (4). For cancer patients with covid-19, the diagnosis and treatment of tumors are often delayed. These people often have a poor prognosis and the mortalitymay increase when the complications cannot be effectively controlled (5).

Currently, conventional treatments for COVID-19 include antiviral drugs, monoclonal antibodies, convalescent plasma, and vaccines. The main effective drugs are Remdesivir, Lopinavir, Ritonavir, Ribavirin, etc. Some drugs should be carefully used such as Chloroquine, Hydroxychloroquine, etc. Due to the adverse reactions (6, 7). Baricitinib, the first oral drug approved by the Food and Drug Administration for the treatment of COVID-19, consists of Paxlovid and Ritonavir (8). Convalescent plasma is primarily used to enhance the patient's immune system or directly enhance the immune response in the patient (9). Vaccines are effective in preventing SARS-CoV-2 infection. Relief of respiratory symptoms and prevention of bacterial infection is especially important for patients with COVID-19. In addition, traditional Chinese medicine has unique effects in conditioning the body and enhancing anti-epidemic ability, which can effectively prevent the deterioration of early and common types of COVID-19 (10).

SARS-CoV-2 has been mutating since the outbreak began. The variants that initially attracted public attention were mainly α-CoVs and δ-CoVs, with the earliest outbreaks occurring in the United Kingdom and India, respectively (11, 12). At present, Omicron, first detected in South Africa, has become a globally predominant strain and its kinship is difficult to determine (13). Omicron is more infectious and more dangerous than delta-CoVs (9) due to the reason that it carries a large number of genetic mutations, for example more than 30 genetic mutations are in the spike protein alone. Thus, it is urgent to develop more targeted vaccines due to the increasing mutant strains (14).

This study analyzed a case of COVID-19 virus shedding for 85 days, including the process of disease detection, diagnosis, and treatment. This case is very special. The patient is an elderly woman who has a long course of disease and also suffers from diabetes and lung-occupying site. The purpose was to provide suggestions for epidemic prevention and control.

## Case Presentation

### Hospital A

The patient is a 65-year-old female, farming, married and pregnant. She has no genetic history, no history of infection such as hypertension, hepatitis and tuberculosis, no history of major trauma, surgery and blood transfusion, no history of food allergy, no history of drug abuse, smoking and drinking. On January 20, 2020, she returned to Chengdu from Wuhan. On January 28, the patient developed a cough without an obvious cause, accompanied by fever, general fatigue, dizziness, and other symptoms for 4 h.

The patient was conscious, with a body temperature of 37.7°C and pulse rate of 89 beats/min. Multiple index tests were performed on the patient (Table 1). Chest CT examination revealed that there was a mass of ~3.9 × 4.2 × 2.7 cm in the middle lobe of the right lung with an edge burr sign, which was considered to be a space-occupying right lung. On January 29, the SARS-CoV-2 nucleic acid test of the patient's rhinitis swab was positive. Combined with the epidemiological history, the diagnosis of COVID-19 was considered. The patient was given two tablets of Kaletra twice a day and interferon atomization twice a day. On January 31, she was transferred to hospital B, a higher-level designated hospital.

**Table 1:** Results of unqualified test indexes.

**Date**	**Project**	**Result**	**Reference range**
January 28	LYMPH%	11.4	20–50
	LYMPH#, × 10^9^/L	0.5	1.1–3.2
	NEUT%	79.8%	40–75
	GGT, U/L	11.9	12–43
	Cr, umol/L	57.1	62–106
	GLU, mmol/L	6.24	0.7–1
	Na+, mmol/L	136.1	137–145
January 29	CEA, ng/mL	8.11	0–5
	CA199, U/mL	51.2	<35
January 30	WBC, × 10^9^/L	3.2	3.5–9.5

*LYMPH%, Lymphocyte ratio; LYMPH#, Lymphocyte count; NEUT%, Neutrophil ratio; GGT, γ-Glutamyltransferase; Cr, Creatinine; GLU, Glucose; Na+, Sodium; CEA, Carcinoembryonic antigen; CA199, Carbohydrate antigen199; WBC, White Blood Cell*.

### Hospital B

**Figure 1:**
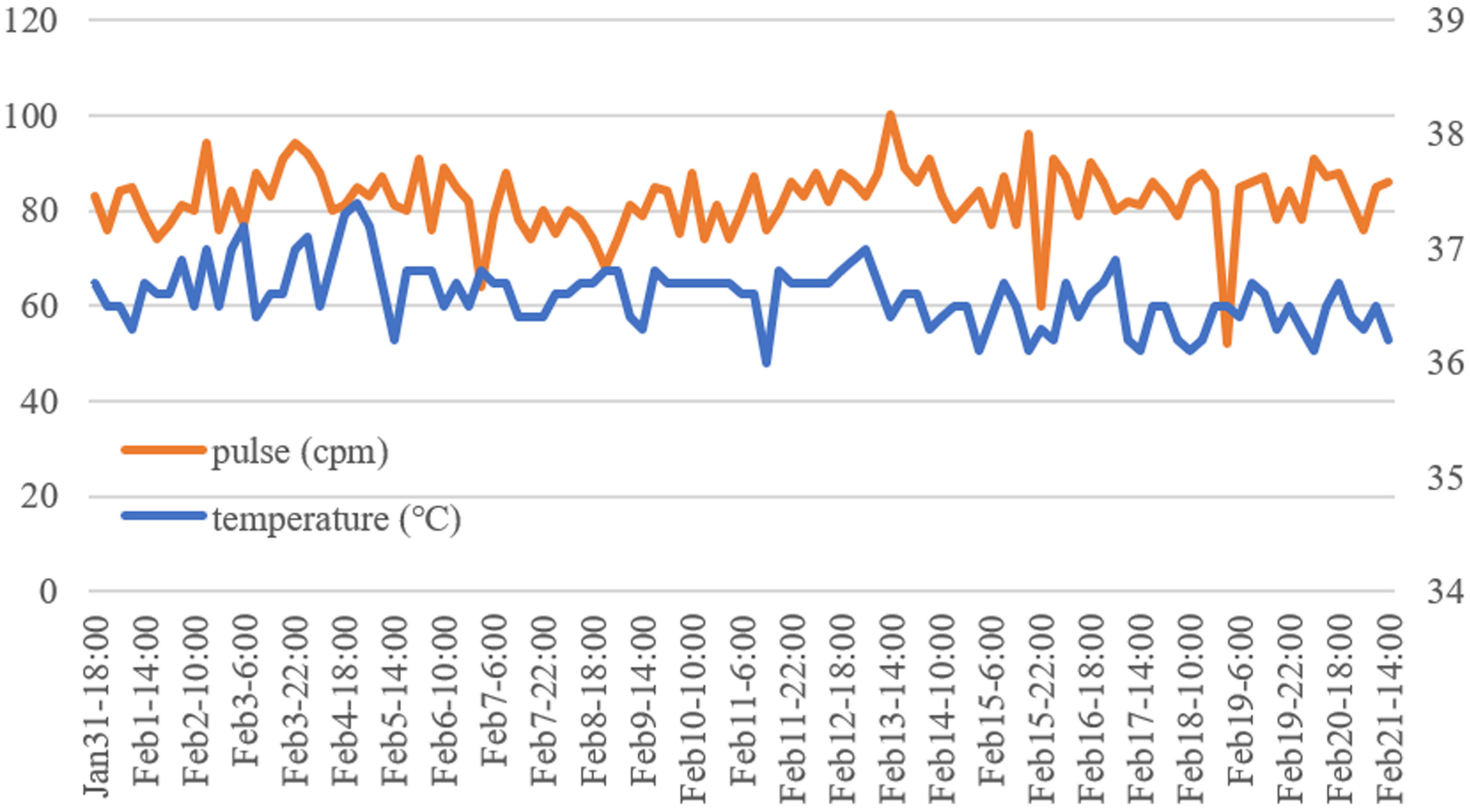
CT images of the patient for 6 days. Each CT chest scan image corresponds to a pulmonary window image and a mediastinal window image. CT on January 31, 2020: A patch of soft tissue density shadow can be seen in the middle lobe of the right lung, showing a “lobular” change. The adjacent pleura and interlobular pleura are stretched, and the right lobe of the liver is nodular and slightly low-density shadow. CT on February 5: Spot-like ground glass density shadows were newly seen in the upper and lower lobes of the right lung and the posterior part of the apex of the left upper lobe. The boundary was blurred. There were also a few fibrous cord shadows in both lungs, adjacent to pleural adhesions. The remaining results are similar to January 31. CT images on and after February 8 showed that the patient's condition continued to improve.

The result of the first CT examination were the same as before, and five more CT examinations were performed during the treatment (Figure 1). After admission, the patient's blood glucose rose repeatedly. The patient was diagnosed with type 2 diabetes mellitus. Her fasting blood glucose value was 5.00 mmol/L, 2 h postprandial was 14.60 mmol/L, and her glycosylated hemoglobin (GHB) was 6.2%. The patient admitted that she had a history of elevated blood glucose, but she did not have further diagnosis or take oral hypoglycemic drugs.

After admission, the patient took 2 capsules kaletra per time orally twice a day to against virus. Lianhuaqingwen granules are taken orally, 3 times a day, 6 g each time, to clear heat and detoxify. On February 2, the patient coughed with a little white sputum. Moxifloxacin hydrochloride 0.4 g was added every day to fight bacterial infection. The patient took orally Acetylcysteine, 0.2 g each time, 3 times a day, to dispel phlegm. On February 3, the patient was diagnosed having cold dampness stagnation of the lung by traditional Chinese medicine physician, so she took Pingweisan, 160 ml per time, three times a day. February 4, aerosol inhalation of α–Interferon 500 IU was introduced twice a day. On February 7, the patient improved. On February 8, the lymphatic count was low. The patient was further improved by taking abido granules, three times a day, one bag each time. On February 11, the patient was diagnosed as phlegm heat stagnation in the lung by traditional Chinese medicine physician. She was given Qingfei Paidu decoction, 160 ml once, three times a day. On February 15, moxifloxacin hydrochloride tablets were discontinued. On February 16, alpha-interferon was discontinued. On February 17, Kaletra and Lianhua Qingwen Granules were discontinued. The patient presented with a toothache and was additionally prescribed ornidazole tablets, 0.5 g, twice a day, for 5 consecutive days. On February 19, the patient had no fever, and the cough and sputum were relieved, thus arbidol granules was discontinued. During the treatment, the doctor timely enlightened the patient's psychology and paid attention to the blood sugar changes. When the patient's appetite was not good, hypoglycemic drugs were temporarily paused, and a diabetic diet was recommended. When the patient improved, the patient was given 0.5 g of metformin extended-release tablets to lower blood sugar after breakfast and dinner.

**Figure 2:**
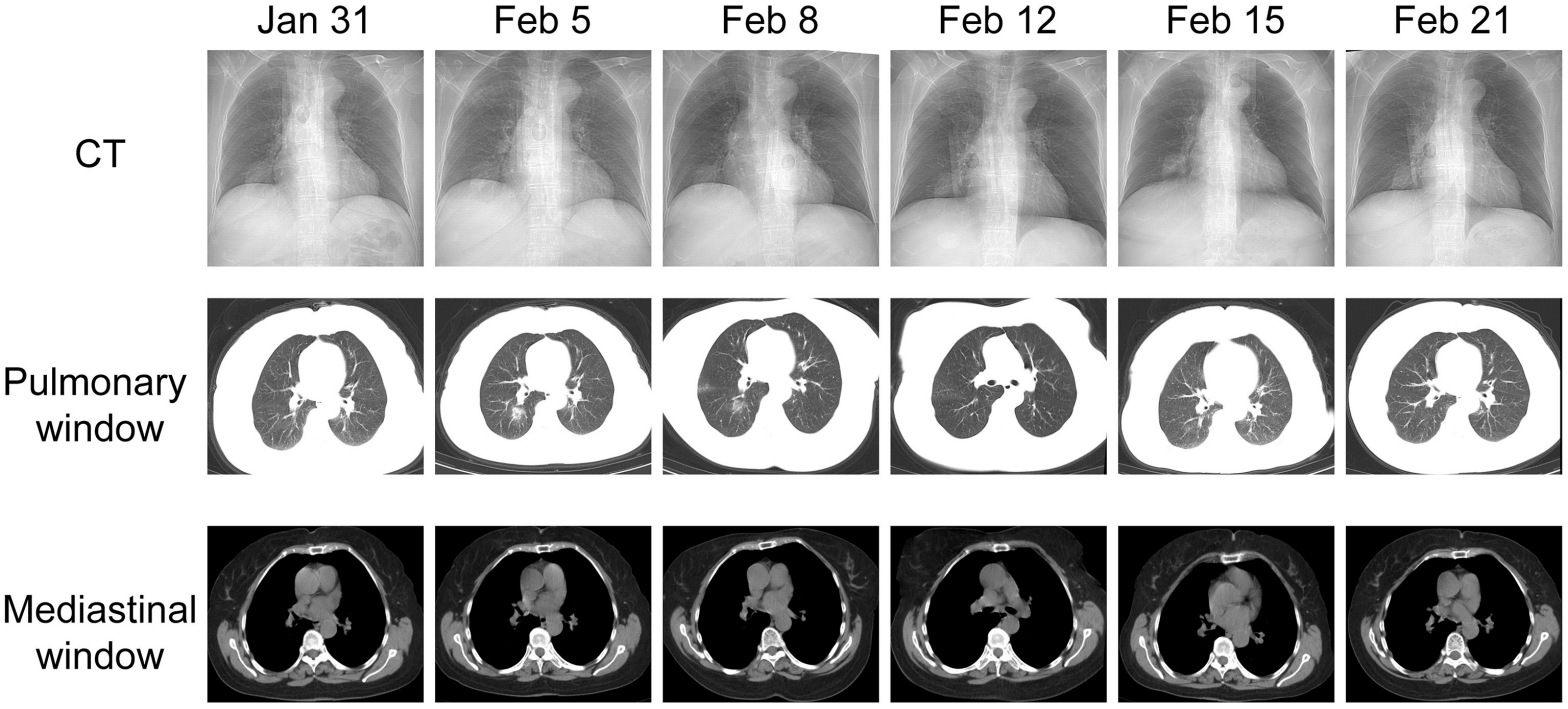
Temperature and pulse changes of the patient. The patient's temperature was normal for more than 15 days. The orange line represents the pulse, corresponding to the data on the left. The blue line represents body temperature, corresponding to the data on the right.

After treatment, the patient's body improved, and her body temperature was normal for more than 15 days (Figure 2). The venous blood test results are shown in Table 2, and the blood gas analysis results are shown in Table 3. On February 19 and February 20, the virus nucleic acid test was rechecked, and the results were all negative. The patient was discharged on February 21.

**Table 2:** Venous blood test results.

**Project**	**January 31**	**February 3**	**February 5**	**February 6 (1)**	**February 6 (2)**	**February 7**	**February 8**	**February 10**	**February 12**	**February 15**	**February 17**	**February 21**	**Reference range**
WBC, × 10^9^/L	N	N	N	3.28	3.28	N	N	N	N	N	N	N	3.5–9.5
RBC, × 10^12^/L	N	5.18											3.8–5.1
LYMPH#, × 10^9^/L	0.85	N	0.94	0.8	1	0.62	0.7	0.69	0.87	0.81	0.96	N	1.1–3.2
LYMPH%		N	N	N	N	12.3	11	15.9	N	18.7	N	N	20–50
NEUT%		N	N	N	N	79.6	81.5	75.8	N	N	N	N	40–75
PDW, fL		N	N	15.2	14.5	14.6	13.9	13.9	15.3	13.7	N	15.3	15.5–18.1
GGT, U/L		N	N	N	N		N	N	N	N	N	50	7–45
TP, g/L		N	N	N	64.4		N	N	N	N	N	N	65–85
ALB, g/L	N	N	N	N	39.9		N	N	N	N	N	N	40–55
TBA, umol/L	34.7												0–15
HS-CRP, mg/L	N	6.77	15.89	16.76	16.15		19.64	26.64	9.15	7.24	7.03	10.2	0–6
Na, mmol/L	N	N	134.6	136.5	N		N	N	N	N	N	N	137–147
P, mmol/L	0.84	N	0.84	0.72	N		0.72	0.74	N	N	N	N	0.85–1.51
CK, U/L			28	28	23		30	28	27	26	20	32	40–200
LDH, U/L			N	N	N		N	N	N	293	N	N	120–250
GLU, mmol/L	7.2	6.2	14.5	14.6	N		8.8	13	9.4	10	N	12.3	3.89–6.11
PT-%	159.4	N	152.7	N	152.7		N	159.4	N	152.7	156	N	70–150
PT-INR	0.87	0.9	0.89	0.93	0.89		0.92	0.87	0.91	0.89	0.88	N	0.95–1.24
Fbg-C, g/L		4.217	4.9	4.028	4.318		4.318	5.936	5.131	N	N	N	2–4
PCT, ng/mL		N	N	N			0.141	0.172	N	N	N	N	0–0.1
2hPG, mmol/L				14.6									3.3–7.8
GHB, %				6.2									4.1–6.0
β2-MG, mg/L	4.7												0.8–2.4

*WBC, White Blood Cell; RBC, red blood cell; LYMPH#, Lymphocyte count; LYMPH%, Lymphocyte ratio; NEUT%, Neutrophil ratio; PDW, Platelet distribution width; GGT, γ-Glutamyltransferase; TP, Total Protein; ALB, albumin; TBA, Total bile acid; HS-CRP, hypersensitive C-reactive protein; Na, Sodium; P, Phosphorus; CK, Creatine Kinase; LDH, lactate dehydrogenase; GLU, Blood glucose; PT-%, prothrombin time activity; PT-INR, Prothrombin International Normalized Ratio; Fbg-C, Fibrinogen concentration; PCT, procalcitonin; 2hPG, 2-h postprandial blood glucose; GHB, glycated hemoglobin; β2-MG, β2-microglobulin; N, normal; blank, The project was not detected on that day*.

**Table 3:** Blood gas analysis results.

**Project**	**February 5**	**February 6**	**February 8**	**February 9**	**February 10**	**February 11**	**February 12**	**February 13**	**February 14**	**Reference** **range**
Ph	N	N	N	N	N	N	N	N	7.47	7.35–7.45
PO_2_, mmHg	N	N	112	76.5	N	N	76.9	77.9	N	80–100
K+, mmol/L	N	N	N	3.44	N	N	N	N	N	3.5–5.6
GLU, mmol/L	14.3	16.2	13.4	11.6	15.8	10.2	11.8	10.3	6.6	3.92–6.11
Lac, mmol/L	2.8	2.92	2.51	2.12	3.17	2.3	2.73	N	N	0.5–2
HCO3act, mmol/L	21.6	N	N	N	N	N	N	N	N	21.7–27.3
THbc, g/L	N	N	N	115	N	N	113	115	110	117–174
Hct, %	N	N	N	34	N	N	33	34	32	35–53
OHb, %	N	N	97.3	N	N	N	N	N	N	94–97

*Ph, Hydrogen ion concentration; PO_2_, partial pressure of oxygen;K+, potassium ion; GLU, Blood glucose; Lac, lactic acid; HCO3act, acture bicarbonate; THbc, Total hemoglobin concentration; Hct, packed cell volume; OHb,Oxyhemoglobin ratio*.

The patient was isolated and observed for 14 days, and the SARS-CoV-2 nucleic acid test was negative for two routine rechecks. Since then, the patient has been isolated at home by herself. During this period, no SARS-CoV-2-infected persons were found around her.

### Hospital C

To further treat the right lung nodule, the patient went to Hospital C on April 13, 2020. Due to a previous history of SARS-CoV-2 infection, she was treated in isolation after admission. The patient had no obvious symptoms. She said that she had lost weight, had blood in her stool for half a year, had constipation for nearly 20 days, and had a loss of appetite. She reported taking oral diabetes drugs for ~2 months.

The patient's neutrophil ratio was 77%, and glycosylated hemoglobin was 6.1%, which was higher than the normal range. The lymphocyte number was 0.96 × 109/L, and the lymphocyte ratio was 14.6%, which was lower than the normal range. The fecal occult blood test was positive. In addition, the patient's tumor markers 1CA50, CEA1, CA199, and CA242 were high. She was treated with ceftizoxime to prevent infection and potassium dehydroandrograpolide succinate for symptomatic treatment. Surprisingly, the patient's two SARS-CoV-2 nucleic acid tests were positive. On April 14, the patient was transferred to Hospital D, a designated hospital.

### Hospital D

Chest CT showed no significant changes in the right middle lobe nodules compared to March 20. There were low-density nodules in the liver during the scan and suspected cysts. Pathological examination of lung puncture material showed adenocarcinoma in the fibrous tissue. Tumor cell immunophenotype: CK7(–), CK20 (+), CDX-2 (+), SATB2 (+), TTF (individual+), and Napsin A (–). Combining the results of morphology and immunohistochemistry, the lesion was diagnosed as intestinal adenocarcinoma metastasis. Prior to this diagnosis, the patient had no previous medical history in the bowel. The patient was unwilling to take intestine examination due to the poor physical condition. The SARS-CoV-2 antibody detection results were IgG+ and IgM–. The patient inhaled 5 million U of alpha-interferon twice a day and received intravenously injected ribavirin twice a day, 0.5 g each time. In addition, the metformin and acarbose were used to control blood glucose. During hospitalization, the patient believed she was seriously ill and the end was coming, thus she was in a negative mood. The psychiatrist diagnosed the patient with anxiety and depression. To raise the patient's spirit, paroxetine 10 mg once a day and tandospirone 5 mg three times a day were given.

On April 21 and April 22, the patient's SARS-CoV-2 nucleic acid test results were all negative. On April 22, the lymphocyte subsets of the patients were reexamined, and the CD4+ count value was 358 cells/UL. Subcutaneous injection of 1.6 mg thymine twice a week enhanced immunity. The patient was discharged on April 23.

### Telephone Follow-Up

Through telephone follow-up, it was learned that after the patient was discharged from the hospital, she actively cooperated with the epidemic management, self-isolated, and performed viral nucleic acid tests many times, and the results were all negative. The patient had a negative attitude toward cancer, but will face it calmly. She doubted that her SARS-CoV-2 nucleic acid test turned positive again, and wondered whether the test result was wrong. The patient was very grateful to the medical workers for their help.

## Discussion

This study analyzed a case of COVID-19 virus shedding for 85 days. The elderly have a higher risk of SARS-CoV-2 infection, often accompanied a variety of complications and higher mortality (15, 16). Patients with T2DM are more susceptible to SARS-CoV-2. T2DM favors the expression of angiotensin-converting enzyme 2 (ACE2) in tissues and promotes the binding of the spike protein of SARS-CoV-2 to ACE2 (17). ACE2 is a plasma membrane protein that is the receptor for the SARS-CoV-2 spike protein (18), mainly expressed in tissues such as the lungs and intestines. In addition, the immunodeficiency of T2DM patients also increases the risk of infection, such as redox stress, metabolic syndrome, inflammation, β-cell dysfunction, etc. (19). On the other hand, SARS-CoV-2 can exert a diabetogenic effect by binding to ACE2 in pancreatic beta cells (20). Although glycemic control is particularly important in reducing the risk of COVID-19 disease and promoting prognosis, there are many influencing factors in observational studies, such as gender, the severity of the disease, etc. The association between hypoglycemic therapy and COVID-19 remains unclear. Market-approved oral antidiabetic drugs, such as the commonly used first-line antidiabetic drug metformin, appear to be safe for COVID-19 patients. However, it is not recommended for patients with COVID-19 to take hypoglycemic drugs when they are seriously ill. Blood glucose should be monitored dynamically and lowered under safe conditions (21–23).

Cancer patients have fragile immune systems and are susceptible to SARS-CoV-2 infection, often with multiple complications (24). Compared with patients with solid organ tumors, patients with malignant tumors are more susceptible. Patients with hematological malignancies, lung cancer, and tumor metastases had a higher proportion of severe events (16). Certain cancer patients, such as cervical cancer patients who need radiation therapy to prolong their lives, cannot stop radiation therapy during COVID-19 treatment. But for many critically ill patients, too much cancer intervention can exacerbate the disease (25). As the case we report here, COVID-19 delays the diagnosis and treatment of cancer. Cancer patients require more oxygen therapy and focus on antiviral, anti-inflammatory, and immune-enhancing treatments (26). Even for cancer patients who have recovered from COVID-19, we encourage enhanced personal protection and testing (25), minimize exposure risks, and extend isolation periods. A decision on surgical intervention must be made after rigorous ethical and clinical evaluation.

After the patient was first diagnosed as SARS-CoV-2-positive, she did not receive chemotherapy for the tumor. In the initial stage, the combined use of interferon and Kaletra showed a certain effect. In Hospital B, the patient was treated with antiviral and anti-infective treatments, and acetylcysteine, Lianhua Qingwen, and other drugs were added for symptomatic and supportive treatment. Acetylcysteine, as a mucolytic agent (27), can alleviate mucus deposition in patients with COVID-19 to a certain extent. Lianhua Qingwen has been proven to inhibit the replication of SARS-CoV-2 and reduce the release of host cytokines. It has a therapeutic effect on COVID-19 patients and is safe (28, 29). Pingwei San, as a dehumidifier, mainly invigoras spleen and stomach. Qingfei Paidu decoction has a good therapeutic effect on early or common COVID-19 patients with wind-cold or cold dampness *in vitro* and blood stasis and heat *in vivo* (10, 30). Traditional Chinese medicine in the field of disease treatment needs to be explored. In addition, the hospital actively controls the blood sugar of patients, strengthens the monitoring of patient signs, and avoids possible metabolic interference with antidiabetic drugs (4). The doctor controlled the patient's blood sugar. The combined treatment of Chinese and Western medicine by the hospital is relatively novel and has shown a certain effect.

Why do we believe the patient has a SARS-CoV-2 relapse rather than reinfection? When the patient's SRS-COV-2 nucleic acid test result was positive again, she did not show symptoms. She was discharged soon after receiving treatment. Due to sample and time reasons, we could not sequence these two viruses, so the possibility of reinfection could not be completely ruled out (31). But, we believed that the patient was less likely to be infected with SARS-CoV-2 again because she had been in isolation since the first recovery and discharge. At that time, China's epidemic prevention and control efforts were very strict. Under the combination of self-isolation and community isolation, the patient only had the opportunity to contact family members and doctors. During this period, multiple nucleic acid tests of the patient were negative, and no COVID-19 patients were found around the patient. We speculate that it may have been the influence of the test sample or method that caused a false negative in the previous test result, rather than a true “cure.” Throat swab nucleic acid testing is convenient, fast and widely used. But the results of RT–PCR are easily affected by factors, such as sample collection, course of the disease, and detection reagents, which result in a false negative. The sensitivity of the throat swab test decreases with time (32). Even if the nucleic acid results of throat swabs were negative, SARS-CoV-2 could still be detected in sputum or stool (33). The patient's first “recovery” may be due to the suppression of the virus by the autoimmune system. However, after discharge, the balance between the immune system and SARS-CoV-2 may be broken due to physical fitness and immunotherapy. Patients with immunodeficiency may take longer to shed the virus (34, 35).

This is a meaningful case in the prevention and treatment of elderly patients with new coronary disease. In this case report, the patient actively got medical attention after symptoms appeared. Although the patients have been transferred to several hospitals, the medical workers all showed meticulous care to the patients, and the development of the disease was controlled on time. It is recommended that the elderly receive regular physical examinations annually and pay attention to their own health.

## Conclusions

We speculate that the tested sample or method caused false-negative results of the viral nucleic acid in the patient. The patient may not be completely cured, but the viral load in her body is low. During the isolation period, the viral load in the patient's body increased, and she finally tested positive again. It is worth noting that for the elderly, especially those with complications or who are prone to diseases, it is recommended to extend the treatment time, and medical staff should pay attention to the psychological state of these patients. In addition, the elderly should pay attention to regular physical examinations and pay attention to their physical condition to avoid the occurrence and metastasis of the disease. At present, the epidemic is still rampant. The prevention and control efforts of the epidemic are still very important. People should pay attention to personal protection, even those who have been “cured.”

## Data Availability Statement

The raw data supporting the conclusions of this article will be made available by the authors, without undue reservation.

## Ethics Statement

The studies involving human participants were reviewed and approved by Medical Ethics Committee of Pidu District People's Hospital of Chengdu (2021 Ethics Clearance No. 236). Written informed consent was obtained from the individual for the publication of any potentially identifiable images or data included in this article.

## Author Contributions

XW sorted out the case and wrote the original manuscript. MS, CY, DP, and XZ collected and summarized the data. FX, JL, MH, LC, HQ, and TL analyzed and explained the patient's test results. XJ designed and supervised this work. All authors have critically revised the article, approved of the final version of the manuscript, and made substantial contribution to the conception of the work.

## Funding

This work was supported by grants from the National Natural Science Foundation of China (Nos. 31870135 and 31600116) and the 1000 Talent Plan of Sichuan Province (No. 980), the Research Fund of Chengdu Medical College (CYZ18-03), and the Research Fund of Non-coding RNA and Drug Discovery Key Laboratory of Sichuan Province (No. FB20-05).

## Conflict of Interest

The authors declare that the research was conducted in the absence of any commercial or financial relationships that could be construed as a potential conflict of interest.

## Publisher's Note

All claims expressed in this article are solely those of the authors and do not necessarily represent those of their affiliated organizations, or those of the publisher, the editors and the reviewers. Any product that may be evaluated in this article, or claim that may be made by its manufacturer, is not guaranteed or endorsed by the publisher.

## Abbreviations

COVID-19: Corona Virus Disease 2019
SARS-CoV-2: Severe Acute Respiratory Syndrome Coronavirus 2
CK7: Cytokeratin 7
CK20: Cytokeratin 20
CDX-2: Caudal-related homeodomain transcription 2
SATB2: Special AT-rich sequence-binding protein 2
TTF-1: Thyriod transcription factor-1.
